# Editorial on Translational Research in Graft-Versus-Host Disease (GVHD) and Graft-Versus-Tumor (GVT) Effect After Allogeneic Hematopoietic Cell Transplantation

**DOI:** 10.3389/fimmu.2022.948720

**Published:** 2022-06-08

**Authors:** Marie Czech, Robert Zeiser, Tomomi Toubai

**Affiliations:** ^1^ Department of Medicine I, Medical Center - University of Freiburg, Faculty of Medicine, University of Freiburg, Freiburg, Germany; ^2^ Faculty of Biology, University of Freiburg, Freiburg, Germany; ^3^ Signaling Research Centre for Biological Signalling Studies (BIOSS) Freiburg and CIBSS – Centre for Integrative Biological Signalling Studies, University of Freiburg, Freiburg, Germany; ^4^ Comprehensive Cancer Center Freiburg (CCCF), Medical Center - University of Freiburg, Faculty of Medicine, University of Freiburg, Freiburg, Germany; ^5^ Department of Internal Medicine III, Division of Hematology and Cell Therapy, Faculty of Medicine, Yamagata University, Yamagata, Japan

**Keywords:** GvHD, allogeneic hematologic stem cell transplantations, JAK - STAT signaling pathway, mesenchymal stem cell, GvT effect

Allogeneic hematopoietic cell transplantation (allo-HCT) is a highly effective treatment for hematological malignancies. However, the effect of allo-HCT is limited by the occurrence of acute graft-*versus*-host disease (GVHD), which is a life-threatening complication of allo-HCT that occurs in up to 50% of the patients (1).

In this Research Topic, the authors cover recent advances of pathophysiology, uncommon manifestations, prevention and treatment strategies for GVHD, as well as approaches to enhance graft-*versus*-tumor (GVT) activity. The authors also discuss the biology of GVHD in mouse models as well as aspects of clinical translation.

GVHD is mediated by alloreactive donor T cells, which recognize MHC molecules as foreign. While alloreactivity is causative for GVHD, it is also necessary to provide the beneficial GVT effect. Huang et al. describe how metabolic modulation of donor T cells could help to reduce GVHD without loss of GVT by targeting glycolysis. The beneficial effects of allo-HCT against malignant tumor cells is not restricted to hematological malignancies as Bates et al. for example explored allo-HCT as platform for the treatment of neuroblastoma. The authors assessed how combining immunocytokine treatment and *ex vivo* activated NK cell infusions could serve as intervention to provide GVT activity in a murine GD2+ neuroblastoma model. E*x vivo* expanded allogeneic T cells may also have activity in anti-viral immunity. Kim et al. describe how antigen specific T cells from donors recovered from Covid-19 can be expanded and manufactured to treat severe disease in partial HLA-matched recipients.

However, even though allo-HCT can be a life-saving intervention, the risk for developing GVHD remains an important point to consider. Research around the pathophysiology and the prevention of GVHD remains a key column for the success of allo-HCT. Due to the prominent role of alloreactive T cells in the induction of GVHD, Jiang et al. review current literature concerning the roles of different T cell subset and their respective cytokine signatures in the context of GVHD and GVT. They also outline preclinical data on the role of these subsets in both, GVHD and GVT effects, and subsequently address strategies to translate these findings to prevent GVHD in patients. Another factor that can be modified to reduce GVHD is the pre-transplant conditioning as it leads to release of danger signals (2). Based on the observation by some investigators that reduced conditioning was associated with higher relapse rates compared to full intensity conditioning (3, 4), Davis et al. elaborate on the potential benefits of repurposing ruxolitinib and venetoclax as pre-transplant medications to improve engraftment and GVT effects while reducing GVHD. Ruxolitinib was developed from the mouse model (5) into clinical application in first treatment series (6) and then in prospective phase III trials for acute and chronic GVHD (7, 8). Venetoclax, sorafenib (9) and other targeted therapies hold promise to enhance the GVT effect.

Besides the topic of strategies to reduce GVHD incidence and severity, this Research topic also covers risk factors that favor the development of GVHD. In their contribution to this series, Khuat et al. investigate how various parameters like the microbiome and high-fat diet, which are addressed using different mouse models, promote and exacerbate GVHD.

The pathophysiology of GVHD is based on a pro-inflammatory environment produced in the target organs, most prominently the skin, liver and gastrointestinal tract (GI). GI manifestations of GVHD however mostly contribute to reduced quality of life and mortality (10) and are mediated by T cells and neutrophils (11). Rayasam and Drobyski review the most foundational studies conducted in animal models that focus on preventing GI-GVHD and how these findings were translated into clinical applications. While the classical GVHD target organs are GI tract, liver and skin, increasing evidence suggests that also other organs such as the kidney, lungs or lymphatic tissues may be affected. In mouse models, T cells and microglia activation were shown to contribute to central nervous system (CNS)-GVHD (12, 13). Clinical studies on neurologic complications after allo-HCT describe the CNS as GVHD target organ (14, 15). However, also infections, vascular events, drug toxicity or other diseases may contribute to neurological symptoms like seizures or cognitive impairment (16). Vinnakota and Zeiser discuss data from mouse studies and clinical reports with a focus on how these findings increased biological understanding of underlying mechanisms and eventually may lead to novel therapy options for CNS-GVHD. Another non-classical clinical manifestation of GVHD is presenting itself as acute kidney injury (AKI). Drugs used as conditioning regimen pre allo-HCT, but also immunosuppressive drugs used to prevent GVHD are known to cause renal damage. However, renal diagnostic criteria are yet to be defined, as AKI often is the result of multiple etiologies (17). Therefore, Miyata et al. describe pathophysiology and management of kidney injury in the context of GVHD.

Mesenchymal Stromal Cell (MSC) products are a promising treatment that is under intensive investigation for GVHD. Kelly and Rasko discuss MSCs and GVHD in their contribution to this Research topic. The activity of MSCs is controversial, as different clinical studies showed responses to MSCs or failed to improve GVHD-related mortality (18, 19) which may be due to MSC preparation, transfer time point, GVHD severity or organ involvement. Murata et al. discuss two commercial MSC products and review clinical studies investigating outcome for patients.

This Research Topic presents recent advances in the field of translational research of GVHD and discuss how these advances are connected to increased mechanistic understanding of the underlying pathophysiology.

## Author Contributions

MC, RZ and TT performed literature research, discussed the articles and wrote the manuscript. All authors contributed to the article and approved the submitted version.

## Funding

This study was supported by the Deutsche Forschungsgemeinschaft, Germany, SFB-1479 – Project ID: 441891347 (P01 to RZ), SFB1160 (RZ).

## Conflict of Interest

RZ received honoraria from Novartis, MNK, Incyte and Sanofi.

The remaining authors declare that the research was conducted in the absence of any commercial or financial relationships that could be construed as a potential conflict of interest.

## Publisher’s Note

All claims expressed in this article are solely those of the authors and do not necessarily represent those of their affiliated organizations, or those of the publisher, the editors and the reviewers. Any product that may be evaluated in this article, or claim that may be made by its manufacturer, is not guaranteed or endorsed by the publisher.
